# Circular RNA circ_0001162 promotes cell proliferation and invasion of glioma via the miR-936/ERBB4 axis

**DOI:** 10.1080/21655979.2021.1932221

**Published:** 2021-05-30

**Authors:** Dexiang Zhou, Xiaofeng Lin, Peng Wang, Yong Yang, Jiantao Zheng, Dong Zhou

**Affiliations:** Department of Neurosurgery, Guangdong Provincial People’s Hospital (Guangdong Academy of Medical Sciences), Guangdong, China

**Keywords:** Circ_0001162, glioma, miR-936, ERBB4

## Abstract

The biological modulatory roles of many circular RNAs (circRNAs) have been validated in glioma. The current study was designed to research the functional mechanism of circ_0001162 in glioma progression. The quantitative real-time polymerase chain reaction (qRT-PCR) was used for assaying the levels of circ_0001162 and microRNA-936 (miR-936). Cell proliferation and colony formation abilities were evaluated via 3-(4, 5-dimethylthiazol-2-y1)-2, 5-diphenyl tetrazolium bromide (MTT) and colony formation assay, respectively. Transwell assay was applied to assess cell migration and invasion. The impact of circ_0001162 on glioma growth in vivo was performed using xenograft tumor assay. The target binding was affirmed via the dual-luciferase reporter and RNA pull-down assays. All protein expression levels were examined via Western blot. Circ_0001162 was an overexpressed circRNA in glioma. Circ_0001162 promoted glioma cell proliferation, colony formation, migration and invasion in vitro. Tumorigenesis of glioma in vivo was also enhanced by circ_0001162. Circ_0001162 could directly target miR-936 and the biological function of circ_0001162 in glioma was related to the inhibition of miR-936. ErbB2 receptor tyrosine kinase 4 (ERBB4) was a direct target of miR-936. Additionally, miR-936 inhibited the glioma development via targeting ERBB4. The miR-936/ERBB4 axis was responsible for the oncogenic role of circ_0001162 in glioma. The effects of circ_0001162 on glioma cells were also associated with the positive regulation of ERBB4. These results indicated that circ_0001162 contributed to the glioma progression via regulating the miR-936/ERBB4 axis, which laid a foundation for the pathomechanism and molecular treatment of glioma.

## Graphical abstract

**Figure:**
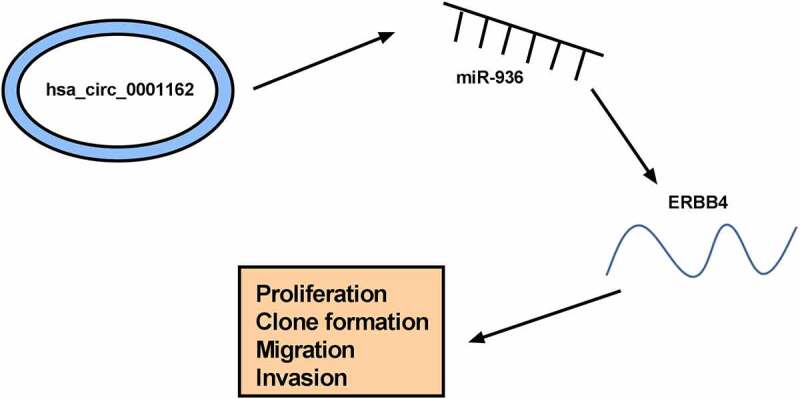

## Introduction

Glioma, a most frequent brain tumor, has become the most aggressive and refractory disease characterized by extremely high mortality [1,2]. Numerous treatments have been developed after the massive efforts by researchers, including surgery, immunotherapy, stem cell therapy and drug therapy [3–5]. The presence of molecular markers related to glioma may provide a new therapeutic perspective for glioma [6].

Circular RNAs (circRNAs) had diagnostic and therapeutic applications in glioma by interacting with miRNAs through miRNA response elements (MREs) (i.e., ‘miRNA sponges’) to reduce the quantity of miRNAs available to target mRNAs and promote mRNA expression [7,8]. For example, circ-0014359 targeted miR-153 to regulate the PI3 signaling pathway to promote glioma development [9] and circ_0034642 contributed to glioma cell proliferation by elevating the BATF3 expression through serving as a miR-1205 inhibitor [10]. Circ_0001162 that was derived from the matrix metalloproteinase 9 (MMP9) gene has been reported as an tumorigenic factor in glioblastoma through targeting miR-124 [11]. Nevertheless, the functional mechanism of circ_0001162 in glioma remains to be discovered.

MiRNAs have pivotal roles in the malignant development and therapeutic management of glioma by repressing the messenger RNA (mRNA) transcripts and protein translation through targeting the 3ʹ-untranslated regions (3ʹUTRs) [12,13]. Xiong et al. explained that miR-320a reduced cell migratory and invasive capacities in glioma via the direct interaction with AQP4 [14]. Jiang et al. stated that miR-214 targeted caspase 1 to induce the inhibitory effects on glioma migration and proliferation [15]. MiR-936 was identified as a tumor repressor factor of glioma via regulating the level of CKS1 [16], but it is unknown about the relation between circ_0001162 and miR-936 in glioma.

ErbB2 receptor tyrosine kinase 4 (ERBB4) is a member of the epidermal growth factor (EGF) receptor family that plays an influential role in the modulation of motor neurons by combining with neuregulins [17]. Yan et al. proclaimed the neuroprotective function of ERBB4 after subarachnoid hemorrhage through activating the YAP/PIK3CB pathway [18]. ERBB4 was also reported to be accountable for the regulation of circ_0074026/miR-1304 axis in the oncogenesis of glioma [19]. The relation among ERBB4, miR-936 and circ_0001162 is unreported.

Herein, we hypothesized that miR-936 was a miRNA target for circ_0001162 and ERBB4 acted as a target gene for miR-936, and circ_0001162 could affect the level of ERBB4 via targeting miR-936 in glioma. The aim of this study was to investigate the regulatory network among circ_0001162, miR-936 and ERBB4 in regulating the initiation and development of glioma.

## Materials and methods

### Tissues source

Glioma tissues were obtained from 30 patients after the glioma resection at Guangdong Provincial People’s Hospital. Histologic subtypes included anaplastic astrocytoma (n = 17), glioblastoma (n = 10), and diffuse astrocytoma (n = 3). Tumor grading was divided into I–II (n = 14) and III–IV (n = 16). All pathophysiological diagnoses were performed by two experienced pathologists. Meanwhile, the normal brain tissues (n = 30) was collected from patients subjected to the excision of brain tissues because of the false-positive result. Before RNA isolation, all specimens were frozen in liquid nitrogen all the time. Each patient has signed the written informed consent for tissue donation. In the ethical aspect, this study was empowered by the Ethics Committee of Guangdong Provincial People’s Hospital.

### Cell culture and treatment

The sources of human glioma cell lines (LN18 and A172) and human astrocytes NHA were American Type Culture Collection (ATCC, Manassas, VA, USA) and ZEYE Biotech (Shanghai, China), respectively. Cells were cultivated with Dulbecco’s modified eagle medium (DMEM) containing 10% Fetal Bovine Serum (FBS) and 1% Antibiotic Solution (Gibco) in 25 cm^2^ culture flasks (Corning Inc., Corning, NY, USA), then placed in a 37°C incubator with humidified air and 5% CO_2_. Cell passage was executed every 3 d with the subculture ratio of 1:4 and the third-generation cells were used in this study. To detect RNA stability, Actinomycin D (2 μg/mL; Millipore, Billerica, MA, USA) was added to LN18 and A172 cells to block transcription for various times.

### Quantitative real-time polymerase chain reaction (qRT-PCR)

Firstly, RNA extraction was administrated via RNAiso Plus (Takara, Beijing, China). For stability analysis, 2 μg RNA was incubated with RNase R (3 U/μg; Epicenter Technologies, Madison, WI, USA) for 30 min at 37°C. The reverse transcription was performed by PrimeScript™ RT Master Mix (Takara) using random or oligo (dt)_18_ primers. The qRT-PCR was conducted through SYBR Green PCR Kit (Applied Biosystems, Foster City, CA, USA). The primers were exhibited as follows: circ_0001162: 5ʹ-GCCAGTTTGCCGGATACAAA-3ʹ (forward) and 5ʹ-TTCTCTCGGTACTGGAAGACG-3ʹ (reverse); miR-936: 5ʹ-TCGGCAGGACAGTAGAGGGAGG-3ʹ (forward) and 5ʹ-CAGTGCGTGTCGTGGAGT-3ʹ (reverse); glyceraldehyde-3-phosphate dehydrogenase (GAPDH): 5ʹ-GGAGCGAGATCCCTCCAAAAT-3ʹ (forward) and 5ʹ-GGCTGTTGTCATACTTCTCATGG-3ʹ (reverse); U6: 5ʹ-CTCGCTTCGGCAGCACATATACT-3ʹ (forward) and 5ʹ-GCTTCACGAATTTGCGTGTC-3ʹ (reverse). The approach of 2^−∆∆Ct^ [20] was employed for analyzing the expression levels of RNA using U6 and GAPDH as the internal references.

### Subcellular localization

The nuclear or cytoplasmic RNA was isolated using the PARIS™ Kit (Invitrogen, Carlsbad, CA, USA) as per the user’s manual. Briefly, cells were lysed with cell fractionation buffer and then centrifugated with the speed of 2000 rpm/min for 10 min. The supernatant with the cytoplasmic fraction was transferred into a sterile RNase-free tube, and the bottom nuclear pellet was lysed with cell disruption buffer. Subsequently, the cytoplasmic or nuclear fraction was passed through a filter cartridge and RNA was eluted by elution solution. Then, the expression detection of circ_0001162, MMP9, GAPDH and U6 was performed by qRT-PCR. U6 and GAPDH were used as the nuclear and cytoplasmic controls, respectively.

### Cell transfection

The overexpression vector pcDNA-circ_0001162 and pcDNA-ERBB4 (circ_0001162 and ERBB4), small interfering RNA (siRNA) for circ_0001162 and ERBB4 (si-circ_0001162 and si-ERBB4), short hairpin RNA (shRNA) vector against circ_0001162 (sh-circ_0001162), miR-936 mimic, miR-936 inhibitor and the negative controls (vector, si-NC, sh-NC, miRNA NC and inhibitor NC) were all derived from GenePharma (Shanghai, China). Cell transfection was implemented via Lipofectamine3000 Reagent (Invitrogen), conforming to the instruction book provided by the manufacturer.

### 3-(4, 5-dimethylthiazol-2-y1)-2, 5-diphenyl tetrazolium bromide (MTT) assay

MTT Cell Proliferation Assay Kit (Invitrogen) was exploited for the detection of cell proliferation after transection of each group. Briefly, 10 μL MTT stock solution (12 mM) was added to each well using a micropipette for 4 h and 150 μL dimethyl sulfoxide (DMSO; Invitrogen) was pipetted to dissolve the formazan. 10 min later, the absorbance of each well was read at 490 nm via the microplate reader.

### Colony formation assay

2 × 10^2^ cells were seeded into a sterile six-well plate, which was gently spun for cell homodisperse. Incubating for about 2 weeks, cell culture was stopped when macroscopic spots were observed; then, cells washing was carried out with phosphate buffered saline (PBS; Gibco), followed by cell fixation via methanol (Sangon, Shanghai, China) for 15 min and cellular staining by crystal violet (Sangon) for 10 min. These stained colonies were counted with 50 cells as one colony under a microscope.

### Transwell assay

2 × 10^4^ transfected cells in the serum-free medium were pipetted into the upper chamber of the transwell 48-well chamber (Corning Inc.), and DMEM medium with 10% FBS was pipetted into the lower chamber as an attractant for LN18 and A172 cells. After incubation at the condition of 37°C and 5% CO_2_ for 24 h, those cells passed through the filter were fastened and dyed in methanol and crystal violet. Especially, it was necessary to envelope the upper transwell chamber using matrigel (Corning Inc.) before cell seeding only in invasion assay but not migration detection. Cell images were acquired by the inverted microscope (Olympus, Tokyo, Japan) at the randomly chosen fields and the numbers were recorded.

### Xenograft tumor assay

Six-week-old BALB/c nude mice (n = 20) were obtained from Vital River Laboratory Animal Technology (Beijing, China), and all procedures were ratified by the Animal Ethics Committee of Guangdong Provincial People’s Hospital. These mice were subcutaneously injected with LN18 cells transfected with circ_0001162 or vector and A172 cells transfected with sh-circ_0001162 or sh-NC, with 5 mice/group. After establishing the xenograft model, we measured the tumor volume (length × width^2^/2) weekly. Twenty-eight days post-injection, all euthanatized mice were dissected and tumor weight was measured by a precision electronic balance. The quantification of circ_0001162 in excised tissues was completed via qRT-PCR.

### Dual-luciferase reporter assay

To construct the recombinational luciferase plasmids, the circ_0001162 and 3ʹUTR of ERBB4 sequences were cloned into the pGL3 vector (Promega, Madison, WI, USA). Luciferase plasmids of wild-type (WT) containing the binding region of miRNA were named as WT-circ_0001162 and WT-ERBB4-3ʹUTR. After the miRNA sites in WT were mutated, MUT-circ_0001162 and MUT-ERBB4-3ʹUTR were generated. To affirm the combination between circ_0001162 or ERBB4 and miRNA, LN18 or A172 cells were, respectively, co-transfected with the above plasmids and miR-936 mimic (miRNA NC) or miR-936 inhibitor (inhibitor NC). Then, the luciferase activity was examined according to the operating instruction of the dual-luciferase reporter system (Promega) after lysing cells.

### RNA pull-down assay

Cell lysates were co-incubated with the biotinylated circ_0001162 probe and M-280 streptavidin magnetic beads (Sigma, St. Louis, MO, USA) for immunoprecipitation. Then, the RNA complexes were collected from the magnetic beads, followed by the immediate quantification of circ_0001162 and miR-936 via qRT-PCR.

### Western blot (WB)

To extract the total protein and detect the protein concentration, Radio Immunoprecipitation Assay buffer (Sigma) and Bradford Protein Assay Kit (Takara) were used in this study. 40 µg proteins were separated on 10% sodium dodecyl sulfate polyacrylamide gel (Invitrogen) for 90 min. Subsequently, proteins on the gel were moved onto the polyvinylidene fluoride (PVDF) membranes (Millipore) and the membranes were sealed in Membrane Blocking Solution (Invitrogen) overnight at 4°C to reduce nonspecific binding. Then, the membranes were submerged in anti-proliferating cell nuclear antigen (anti-PCNA; Abcam, Cambridge, UK, ab18197, 1:1000), anti-matrix metalloproteinase-3 (anti-MMP-3; Abcam, ab53015, 1:1000), anti-MMP-9 (Abcam, ab38898, 1:1000), anti-ERBB4 (Abcam, ab32375, 1:1000) and anti-GAPDH (Abcam, ab9485, 1:3000) diluted by PBS with 0.05% Tween (PBST) at 25°C. 4 h later, the PVDF membranes were washed by PBST three times and incubated with anti-rabbit IgG-HRP (Abcam, ab205718, 1:5000) for 45 min. Following the washing of the membranes by PBST again, the chromogenic reaction was executed via the ECL HRP Substrate Kit (Millipore) and the ERBB4 expression analysis was conducted via ImageLab software version 4.1 (NIH, Bethesda, MD, USA).

### Statistical analysis

Data collected from three experiments were expressed as the mean ± standard deviation (SD), followed by the statistical analysis by SPSS 22.0 and graph plotting via GraphPad Prism 7. The difference comparison was carried out using Student’s t-test for two groups and one-way analysis of variance (ANOVA) followed by Tukey’s test for multiple groups. It was significant of difference if P < 0.05.

## Results

### Circ_0001162 was highly expressed in glioma

**Figure 1.**
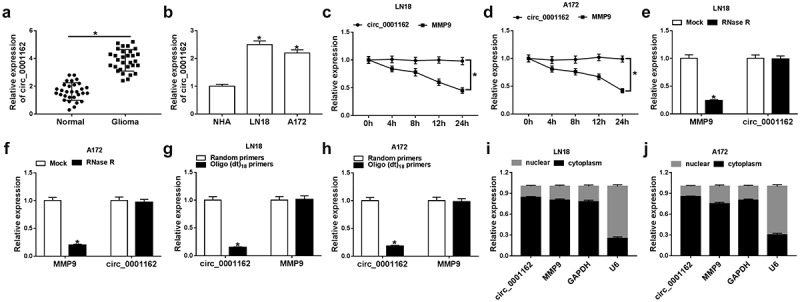
Circ_0001162 was highly expressed in glioma. (a–b) The detection of circ_0001162 by qRT-PCR was performed in glioma samples (a) and cells (b). (c–f) Circ_0001162 stability was determined via qRT-PCR following the treatment of Actinomycin D (c–d) or RNase R (e–f). (g–h) The analysis of circ_0001162 was implemented via qRT-PCR using the reverse transcription products with random or oligo (dt)_18_ primers. (i–j) Circ_0001162 localization was examined via qRT-PCR in LN18 and A172 cells. *P < 0.05

The different regulatory roles and molecular mechanism of circRNAs have been elucidated in glioma. The purpose of this study is to explore what role circ_0001162 plays and how it works. Circ_0001162 was hypothesized as a miR-936 inhibitor to regulate the expression of ERBB4 in glioma progression. Through a series of cellular experiments and target exploration, we confirmed that circ_0001162/miR-936/ERBB4 axis was involved in the regulation of glioma development. Firstly, the expression level of circ_0001162 was determined in glioma. The qRT-PCR analysis revealed that the circ_0001162 level was up-regulated in glioma tissues by contrast with those normal brain tissues (Figure 1(a)). Similarly, the higher expression level of circ_0001162 was observed in LN18 and A172 cells by contrast to NHA cells (Figure 1(b)). The stability analysis indicated that the treatment of Actinomycin D (Figure 1(c–d)) and RNase R (Figure 1(e–f)) did not affect the circ_0001162 expression, while linear MMP9 level was remarkedly decreased. Due to the characteristic of circRNAs without 3ʹ-polyadenylated tails, the random primers or oligo (dt)_18_ primers (combined with the 3ʹ-polyA tail) in the reverse transcription were used to affirm the presence of circ_0001162. The date manifested that circ_0001162 was almost undetectable in the existence of oligo (dt)_18_ primers (Figure 1(g–h)). Additionally, localization detection exhibited that abundant circ_0001162 and MMP9 were found in the cytoplasm by comparison with GAPDH (control of cytoplasm) and U6 (control of nucleus) (Figure 1(i–j)). Collectively, we affirmed the aberrant overexpression of circ_0001162 in glioma.

### Circ_0001162 facilitated the tumorigenesis of glioma in vitro and in vivo

**Figure 2.**
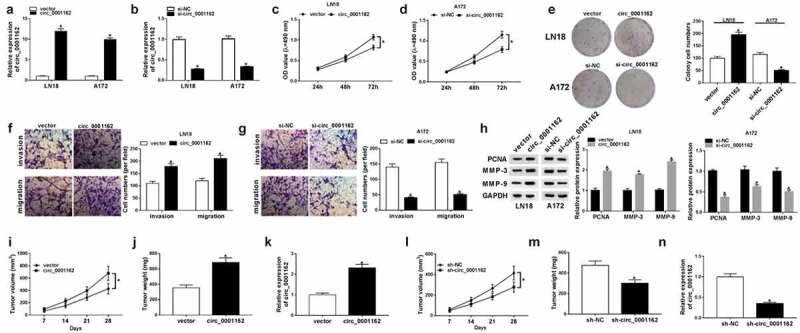
Circ_0001162 facilitated the tumorigenesis of glioma in vitro and in vivo. (a–b) The qRT-PCR analysis of circ_0001162 was performed in LN18 and A172 cells transfected with circ_0001162, si-circ_001162 or their controls. (c–g) The effect of circ_0001162 overexpression (in LN18 cells) or knockdown (in A172 cells) on glioma progression was researched by cell proliferation using MTT (c–d), colony formation by colony formation assay (e) and migration/invasion via transwell assay (f–g). (h) The protein levels of PCNA, MMP-3 and MMP-9 were detected using WB. (i–n) Tumor volume, weight and circ_0001162 expression of glioma were measured after circ_0001162 up-regulation (i–k) or knockdown (l–n). *P < 0.05

On account of the circ_0001162 dysregulation in glioma, we used circ_0001162 overexpression or knockdown to research the role of circ_0001162 in glioma. The results of qRT-PCR demonstrated that circ_0001162 (Figure 2(a)) or si-circ_001162 (Figure 2(b)) transfection in LN18 and A172 cells only exerted the stimulation or inhibition on circ_0001162 expression compared with vector and si-NC controls, respectively. The proliferation ability of LN18 cells transfected with circ_0001162 was enhanced through the comparison with vector group (Figure 2(c)) while circ_0001162 knockdown decreased the absorbance of A172 cells (Figure 2(d)) after the administration of MTT assay. Additionally, the colony number (Figure 2(e)), migrated and invaded cells (figure 2(f–g)) were increased in circ_0001162 group of LN18 cells but reduced in si-circ_0001162 group of A172 cells in contrast to their relative controls. Western blot also revealed that circ_00011162 overexpression upregulated the protein levels of proliferation marker PCNA and metastasis-related MMP-3/MMP-9 in LN18 cells, while circ_0001162 knockdown incurred the downregulation of these proteins in A172 cells (Figure 2(h)). The knockdown of circ_0001162 also impeded cell proliferation, migration and invasion in LN18 cells, while overexpression of circ_0001162 resulted in the promoting effects on these cellular behaviors (Supplementary Figure 1). To validate the carcinogenic function of circ_0001162 in glioma again, xenograft in vivo assay was conducted. As the presentation of Figure 2(i–j), tumor volume and weight were boosted after overexpression of circ_0001162. The circ_0001162 level was higher in tumor of circ_001162 group than that of vector group (Figure 2(k)). On the contrary, our results showed the decreased tumor volume (Figure 2(l)) and weight (Figure 2(m)) triggered by circ_001162 knockdown in vivo, along with the inhibition of circ_0001162 expression (Figure 2(n)). Altogether, our assays identified that circ_0001162 promoted the glioma progression.

### Circ_0001162 directly targeted miR-936

**Figure 3.**
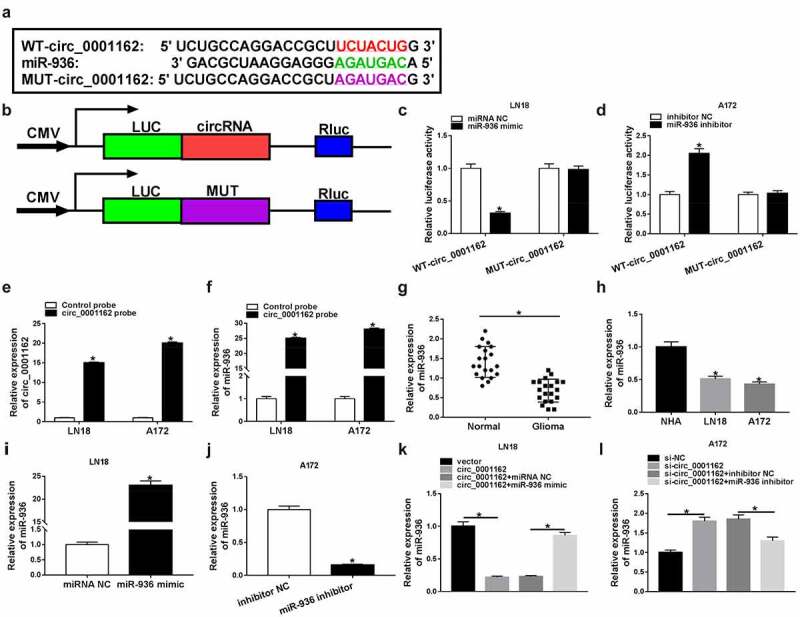
Circ_0001162 directly targeted miR-936. (a) CircRNA interactome was applied for miRNA target prediction of circ_0001162. (b–d) The target relation between circ_0001162 and miR-936 was verified via the dual-luciferase reporter assay. (e) The efficiency of circ_0001162 probe was evaluated via qRT-PCR. (f) The miR-936 level was measured through qRT-PCR in RNA pull-down assay with biotinylated circRNA. (g–h) The detection of miR-936 in glioma tissues and cells was carried out with qRT-PCR. (i–j) The transfection efficiencies of miR-936 mimic (in LN18 cells) and miR-936 inhibitor (in A172 cells) were estimated by qRT-PCR. (k–l) The qRT-PCR was implemented for miR-936 detection in LN18 cells transfected with circ_0001162, circ_0001162+ miR-936 mimic or matched controls and A172 cells with transfection of si-circ_0001162, si-circ_0001162+ miR-936 inhibitor or relative controls. *P < 0.05

The issued reports have indicated that miRNAs can serve as the targets of circRNAs [21,22], then we assumed that circ_0001162 could target miRNA to regulate glioma development. Bioinformatics analysis of circRNA interactome exhibited that the target bites between circ_0001162 and miR-936 (Figure 3(a)). We constructed the luciferase plasmids by inserting the circ_0001162 sequence into the downstream of luciferase reporter gene using MUT-circ_0001162 (the target sites of miR-936 were mutated) as the control (Figure 3(b)). As the data of dual-luciferase reporter assay shown in Figure 3(c–d), miR-936 overexpression inhibited the relative luciferase intensity of LN18 cells transfected with WT-circ_0001162 and miR-936 inhibitor led to the promotion of WT-circ_0001162 group in A172 cells instead of MUT-circ_0001162 groups, contrasted to miRNA NC and inhibitor NC groups, respectively. Besides, RNA pull-down assay displayed that circ_0001162 expression was heightened by biotinylated circ_0001162 probe and miR-936 was also pulled down by this probe (Figure 3(e–f)). In addition, miR-936 level was signally declined in glioma tissues relative to normal tissues (Figure 3(g)) and LN18/A172 cells compared with NHA cells (Figure 3(h)). After identifying the overexpression effect of miR-936 mimic in LN18 cells (Figure 3(i)) and suppressive impact of miR-936 inhibitor in A172 cells (Figure 3(j)) on the level of miR-936, we found the repression (enhancement) of miR-936 caused by circ_0001162 overexpression (knockdown) in LN18 (A172) cells was ameliorated by transfection of miR-936 mimic (inhibitor) (Figure 3(k–l)). Above data manifested the target relation between circ_0001162 and miR-936.

### The oncogenic role of circ_0001162 in glioma cells was owed to miR-936

**Figure 4.**
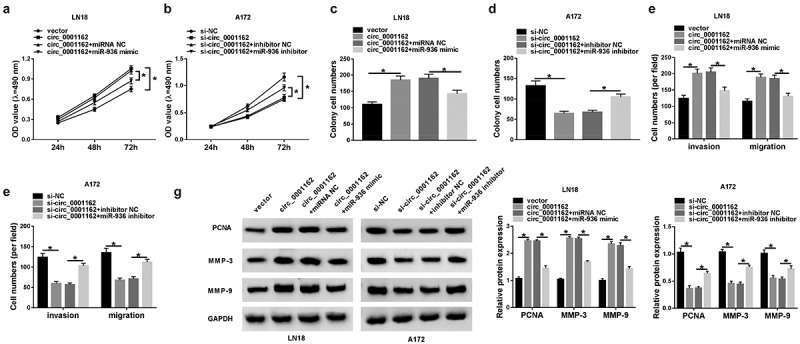
The oncogenic role of circ_0001162 in glioma cells was owed to miR-936. Transfection ofcirc_0001162, circ_0001162+ miR-936 mimic in LN18 cells and si-circ_0001162, si-circ_0001162+ miR-936 inhibitor in A172 cells was performed, including their respective controls. (a–b) The proliferation evaluation was carried out through MTT assay. (c–d) Colony formation analysis was administrated using colony formation assay. (e–f) The determination of cell migration and invasion was conducted via transwell assay. (g) WB was used for the expression analysis of PCNA, MMP-3 and MMP-9. *P < 0.05

The rescue experiments were used to prove whether the function of circ_0001162 in glioma depended on miR-936. MTT assay presented that the circ_0001162-induced promotion of proliferation ability in LN18 cells (Figure 4(a)) and si-circ_0001162-motivated inhibition of that in A172 cells (Figure 4(b)) were, respectively, relieved following the introduction of miR-936 mimic or inhibitor. Also, the effects of circ_0001162 and si-circ_0001162 transfection on colony formation were counteracted by miR-936 overexpression and down-regulation separately (Figure 4(c–d)). Moreover, co-transfection of circ_0001162 and miR-936 mimic abrogated the increase of migrated and invaded cell numbers caused by circ_0001162 in LN18 cells (Figure 4(e)) while miR-936 inhibitor reverted the suppression of migration and invasion in A172 cells transfected with si-circ_0001162 (Figure 4(f)). In addition, WB suggested that circ_0001162 regulated PCNA, MMP-3 and MMP-9 protein levels by the negative regulation of miR-936 (Figure 4(g)). Meanwhile, si-circ_0001162 with miR-936 inhibitor transfection in LN18 cells and circ_0001162 with miR-936 mimic transfection in A172 cells also confirmed the regulation of circ_0001162/miR-936 signal axis in glioma progression (Supplementary Figure 2). Overall, the promoting function of circ_0001162 in glioma progression was ascribed to the negative regulation of miR-936.

### MiR-936 acted as a tumor repressor of glioma via targeting ERBB4

**Figure 5.**
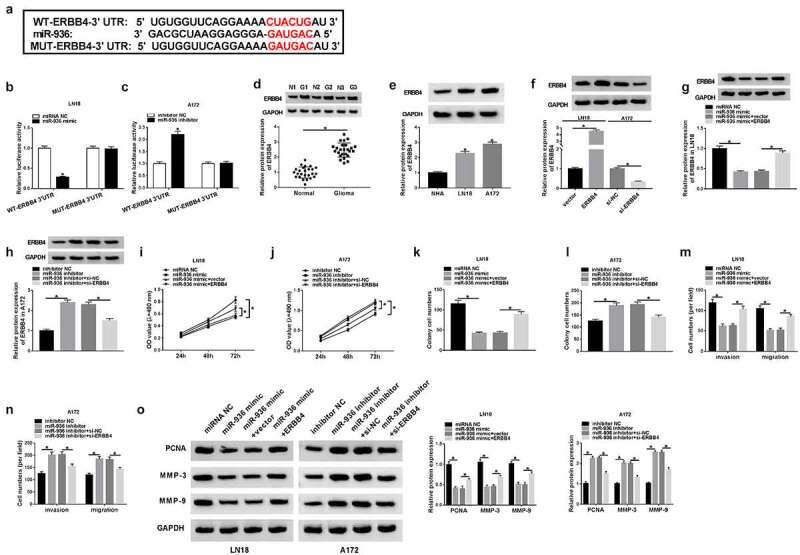
MiR-936 acted as a tumor repressor of glioma via targeting ERBB4. (a) Targetscan exhibited the binding of ERBB4 3ʹUTR and miR-936. (b–c) Whether ERBB4 could bind to miR-936 was verified using dual-luciferase reporter assay. (d–f) WB was administrated to assay the ERBB4 protein level in glioma tissues (d), LN18/A172 cells (e) and the transfection efficiency of ERBB4 and si-ERBB4 (f). (g–h) ERBB4 protein expression was detected via WB after transfection of miR-936 mimic, miR-936 mimic+EREBB4 or controls in LN18 cells (g) and miR-936 inhibitor, miR-936 inhibitor+si-ERBB4 or controls in A172 cells (h). (i–n) Cellular proliferation (i–j), colony formation (k–l) and migration or invasion (m–n) were severally evaluated using MTT, colony formation assay and transwell assay. (o) PCNA, MMP-3 and MMP-9 protein expression detection was performed by WB. *P < 0.05

Our prediction result of Targetscan showed the binding bites between 3ʹUTR of ERBB4 and miR-936 (Figure 5(a)), and the subsequent dual-luciferase reporter assay affirmed the combination between ERBB4 and miR-936 as Figure 5(b–c) depicted. Notably, ERBB4 protein level was enhanced in glioma samples and cells (LN18 and A172) in comparison with their normal controls (Figure 5(d–e)). The results of WB indicated that ERBB4 transfection brought about the increase of ERBB4 protein expression in LN18 cells while the effect of si-ERBB4 was opposite in A172 cells (Figure 5(f)). Whereafter, our rescued experiments manifested that ERBB4 protein downregulation was induced by miR-936 mimic (Figure 5(g)) and expression promotion was caused by miR-936 inhibitor (Figure 5(h)), whereas these effects were alleviated after ERBB4 up-regulation and under-expression, respectively, in glioma cells. Overexpression of miR-936 downregulate the level of ERBB4 to generate the suppressive influences on the proliferation (Figure 5(i–j)), colony formation (Figure 5(k–l)) and migration or invasion (Figure 5(m–n)) of LN18 cells, while miR-936 inhibition incurred the accelerative effects on these cellular behaviors via elevating ERBB4 expression. The results from WB assay exhibited that miR-936 overexpression inhibited PCNA, MMP-3 and MMP-9 protein expression by downregulating ERBB4 while these protein levels were increased by miR-936 inhibitor to upregulate ERBB4 (Figure 5(o)). These results hinted that miR-936 played an anti-cancer role in glioma cells by targeting ERBB4.

### Circ_0001162 contributed to the glioma progression through the regulation of miR-936/ERBB4

**Figure 6.**
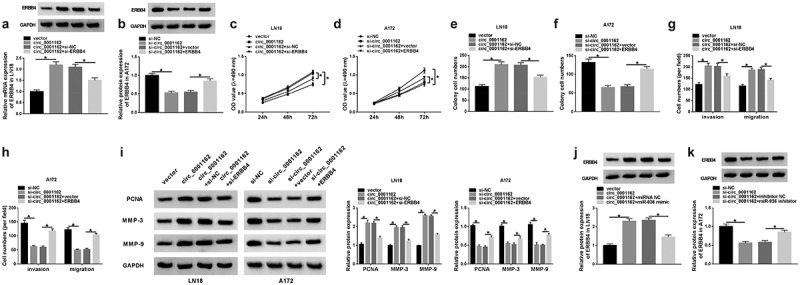
Circ_0001162 contributed to the glioma progression through the regulation of miR-936/ERBB4. (a–b) ERBB4 protein quantification by WB was carried out in LN18 cells with transfection of circ_0001162, circ_0001162+ si-ERBB4 or their controls (a) and A172 cells with transfection of si-circ_0001162, si-circ_0001162+ ERBB4 or respective controls (b). (c–f) The examination of cell proliferation using MTT (c–d) and colony formation via colony formation assay (e–f) was executed in the above groups. (g–h) Transwell assay was adopted for assessing migration and invasion. (i) The measurement of PCNA, MMP-3 and MMP-9 protein expression was completed via WB. (j–k) WB was exploited for estimating the effect of the circ_0001162/miR-936 axis on the ERBB4 protein level. *P < 0.05

To explore the relation between circ_0001162 and ERBB4, transfection of circ_0001162, circ_0001162+ si-ERBB4 in LN18 cells and si-circ_0001162, si-circ_0001162+ ERBB4 in A172 cells were conducted, including their controls. As exhibited in Figure 6(a–b), circ_0001162 could positively regulate the protein expression of ERBB4 and ERBB4 knockdown or overexpression separately restored the effects of circ_0001162 or si-circ_0001162 on ERBB4 level. Moreover, the improvement of circ_0001162 up-regulation on cell proliferation in LN18 cells as well as the restriction of circ_0001162 inhibition in A172 cells were ameliorated by the positive modulation of ERBB4 (Figure 6(c–d)). The same phenomenon was viewed in the detection of colony formation (Figure 6(e–f)) and migration or invasion (Figure 6(g–h)). ERBB4 knockdown also assuaged the circ_0001162-induced protein upregulation of PCNA, MMP-3 and MMP-9 in LN18 cells, and the reversal of ERBB4 overexpression for the inhibition of circ_0001162 was also observed in A172 cells (Figure 6(i)). The results of co-transfection with si-circ_0001162+ ERBB4 in LN18 cells and circ_0001162+ si-ERBB4 in A172 cells further validated that the effects of circ_0001162 on glioma cell proliferation, migration and invasion were associated with the positive regulation of ERBB4 (Supplementary Figure 3). Furthermore, we found that the regulatory impact of circ_0001162 on ERBB4 protein level was achieved by targeting miR-936 (Figure 6(j–k)). Taken together, circ_0001162 promoted the evolvement of glioma by the miR-936/ERBB4 axis.

## Discussion

CircRNAs are highly conserved and stable ncRNAs possessing the modulatory importance in neurological diseases, including glioma [23,24]. Nevertheless, the functional mechanisms of many circRNAs have not been completely addressed. This study proved that circ_0001162 accelerated the tumorigenesis of glioma via the mediation of the miR-936/ERBB4 axis, providing a novel regulatory network about the pathological mechanism of glioma with circ_0001162 as an object of study.

A research of circ_0046701 indicated its pro-carcinoma action in glioma through enhancing the level of ITGB8 by targeting miR-142-3p [25]. Jin et al. asserted that circHIPK3 exerted the promoted influence on glioma occurrence through directly targeting miR-654 to control the IGF2BP3 expression and it could be used as a prognostic marker [26]. The effect of circ_0074362 on glioma progression was achieved through depending on the miR-1236-3p/HOXB7 axis [27]. Herein, our experiments in vitro confirmed that circ_0001162 could promote glioma cell proliferation, colony formation, migration and invasion. Meanwhile, tumor growth in vivo was also facilitated by circ_0001162. These findings were consistent with the previous report of circ MMP9 [11]. In addition, circ_0001162 was reported to aggravate the metastasis of oral squamous cell carcinoma [28]. It also served as a promoter for migration and invasion in osteosarcoma by the miR-1265/CHI3L1 axis [29]. The oncogenic function of circ_0001462 in glioma was in coincident with that in these carcinomas.

Through the online prediction of circRNA interactome and the experimental validation of dual-luciferase reporter/RNA pull-down assays, miR-936 was considered as a miRNA target of circ_0001162. Moreover, the regulation of circ_0001162 in glioma was attributed to the inhibition of miR-936. Zhou et al. announced that miR-936 targeted E2F2 to repress cell proliferation and invasion in non-small cell lung cancer [30] and Li et al. declared that miR-936 acted as a suppressor of epithelial ovarian cancer aggressiveness through blocking the FGF2-mediated PI3K/AKT signaling pathway [31]. Similarly, we identified that miR-936 was an anti-tumor factor in glioma by targeting ERBB4.

Interestingly, the molecular circRNA/miRNA/mRNA network has been explored in various types of human tumors, such as colorectal cancer [32], hepatocellular carcinoma [33] and laryngeal squamous cell carcinoma [34]. In addition, circZNF609/miR-134-5p/BTG-2 axis and circ_0076248/miR-181a/SIRT1 axis have also been disclosed in glioma [35,36]. The previous publications in Bioengineered also affirmed the involvement of circRNA/miRNA/mRNA axis in different tumors. For example, circ-LRP6 functioned as an oncogenic regulator in esophageal squamous cell carcinoma by targeting the miR-182/Myc signaling [37] and circ-ZNF609 facilitated the development of cervical cancer through the miR-197-3p-mediated E2F6 upregulation [38]. Also, circ_0000117 promoted cell proliferation and invasion in gastric cancer via regulating STAT3 expression by competitively binding to miR-337-3p [39]. However, the investigation of circRNA in glioma is little found in Bioengineered. In the current report, we first discovered that circ_0001162 could affect the oncogenesis of glioma by targeting miR-936 to modulate the level of ERBB4. Compared to the previous studies, this study provided a specific circ_0001162/miR-936/ERBB4 signal axis in glioma progression and might lay the great foundation for circRNA research of glioma in the future.

This study remains some limitations. For instance, whether circ_0001162 can regulate the expression of other Erb family member is unclear. We have predicted that ERBB2 also contained the binding sites for miR-936. It is possible that circ_0001162 can target miR-936 to affect the ERBB2 level, and it needs further validation in our future study. In addition, it will be interesting to explore the downstream signaling pathway of circ_0001162/miR-936/ERBB4 axis and the functional mechanism of circ_0001162 can be better understood.

## Conclusion

**Figure 7.**
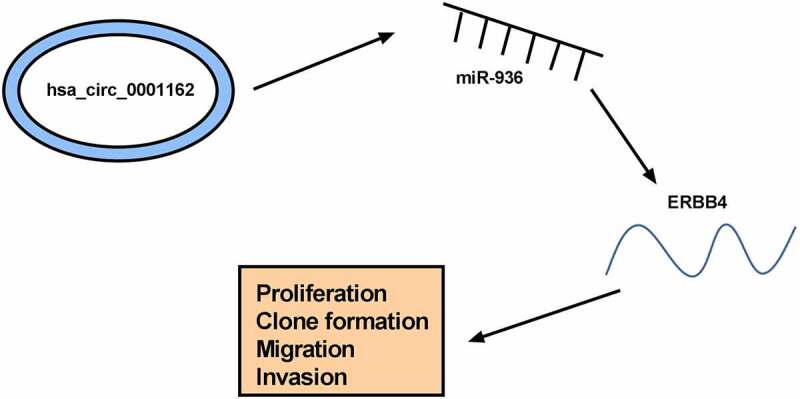
The graphical abstract of this study

The current findings revealed that circ_0001162 targeted miR-936 to regulate the expression of ERBB4, consequently promoting cell malignant behaviors (proliferation, colony formation, migration and invasion) to act as a proto-oncogene in glioma (Figure 7). The discovery of the circ_0001162/miR-936/ERBB4 network might provide a ponderable insight into the underlying biological peculiarity of glioma. Glioma treatment might be improved using circ_0001162 as a therapeutic target.

## Supplementary Material

Supplemental Material

## Highlights

1. The overexpression of circ_0001162 is found in glioma tissues and cells.
2. Circ_0001162 contributes to the tumorigenesis of glioma in vitro and in vivo.
3. Circ_0001162 can directly target miR-936 and miR-936 targets ERBB4.
4. The oncogenic role of circ_0001162 in glioma is attributed to the miR-936/ERBB4 axis.

## References

[1] Gladson CL, Prayson RA, Liu WM. The pathobiology of glioma tumors. Annu Rev Pathol. 2010;5:33–50. 19737106 10.1146/annurev-pathol-121808-102109 PMC2887670

[2] Ostrom QT, Gittleman H, Truitt G, et al. CBTRUS statistical report: primary brain and other central nervous system tumors diagnosed in the United States in 2011-2015. Neuro Oncol. 2018;20:iv1–iv86. 30445539 10.1093/neuonc/noy131 PMC6129949

[3] Van Ierschot F, Bastiaanse R, Miceli G. Evaluating spelling in glioma patients undergoing awake surgery: a systematic review. Neuropsychol Rev. 2018;28:470–495. 30578451 10.1007/s11065-018-9391-7

[4] Lin L, Cai J, Jiang C. Recent advances in targeted therapy for glioma. Curr Med Chem. 2017;24:1365–1381. 28019637 10.2174/0929867323666161223150242

[5] Chen TC, da Fonseca CO, Schonthal AH. Intranasal perillyl alcohol for glioma therapy: molecular mechanisms and clinical development. Int J Mol Sci. 2018;19:3905. 10.3390/ijms19123905 PMC6321279 30563210

[6] Zeng T, Cui D, Gao L. Glioma: an overview of current classifications, characteristics, molecular biology and target therapies. Front Biosci (Landmark Ed). 2015;20:1104–1115. 25961548 10.2741/4362

[7] Liu J, Zhao K, Huang N, et al. Circular RNAs and human glioma. Cancer Biol Med. 2019;16:11–23. 31119043 10.20892/j.issn.2095-3941.2018.0425 PMC6528446

[8] Ren S, Lin P, Wang J, et al. Circular RNAs: promising molecular biomarkers of human aging-related diseases via functioning as an miRNA sponge. Mol Ther Methods Clin Dev. 2020;18:215–229. 32637451 10.1016/j.omtm.2020.05.027 PMC7326721

[9] Shi F, Shi Z, Zhao Y, et al. CircRNA hsa-circ-0014359 promotes glioma progression by regulating miR-153/PI3K signaling. Biochem Biophys Res Commun. 2019;510:614–620. 30745107 10.1016/j.bbrc.2019.02.019

[10] Yang M, Li G, Fan L, et al. Circular RNA circ_0034642 elevates BATF3 expression and promotes cell proliferation and invasion through miR-1205 in glioma. Biochem Biophys Res Commun. 2019;508:980–985. 30551880 10.1016/j.bbrc.2018.12.052

[11] Wang R, Zhang S, Chen X, et al. EIF4A3-induced circular RNA MMP9 (circMMP9) acts as a sponge of miR-124 and promotes glioblastoma multiforme cell tumorigenesis. Mol Cancer. 2018;17:166. 30470262 10.1186/s12943-018-0911-0 PMC6260852

[12] Palumbo S, Miracco C, Pirtoli L, et al. Emerging roles of microRNA in modulating cell-death processes in malignant glioma. J Cell Physiol. 2014;229:277–286. 23929496 10.1002/jcp.24446

[13] Zhou Q, Liu J, Quan J, et al. MicroRNAs as potential biomarkers for the diagnosis of glioma: a systematic review and meta-analysis. Cancer Sci. 2018;109:2651–2659. 29949235 10.1111/cas.13714 PMC6125451

[14] Xiong W, Ran J, Jiang R, et al. miRNA-320a inhibits glioma cell invasion and migration by directly targeting aquaporin 4. Oncol Rep. 2018;39:1939–1947. 29484417 10.3892/or.2018.6274

[15] Jiang Z, Yao L, Ma H, et al. miRNA-214 inhibits cellular proliferation and migration in glioma cells targeting caspase 1 involved in pyroptosis. Oncol Res. 2017;25:1009–1019. 28244850 10.3727/096504016X14813859905646 PMC7840997

[16] Wang D, Zhi T, Xu X, et al. MicroRNA-936 induces cell cycle arrest and inhibits glioma cell proliferation by targeting CKS1. Am J Cancer Res. 2017;7:2131–2143. 29218238 PMC5714743

[17] Lopez-Font I, Sogorb-Esteve A, Javier-Torrent M, et al. Decreased circulating ErbB4 ectodomain fragments as a read-out of impaired signaling function in amyotrophic lateral sclerosis. Neurobiol Dis. 2019;124:428–438. 30594809 10.1016/j.nbd.2018.12.021

[18] Yan F, Tan X, Wan W, et al. ErbB4 protects against neuronal apoptosis via activation of YAP/PIK3CB signaling pathway in a rat model of subarachnoid hemorrhage. Exp Neurol. 2017;297:92–100. 28756200 10.1016/j.expneurol.2017.07.014

[19] Chen M, Liu X, Xie P, et al. Circular RNA circ_0074026 indicates unfavorable prognosis for patients with glioma and facilitates oncogenesis of tumor cells by targeting miR-1304 to modulate ERBB4 expression. J Cell Physiol. 2020;235:4688–4697. 31643077 10.1002/jcp.29347

[20] Livak KJ, Schmittgen TD. Analysis of relative gene expression data using real-time quantitative PCR and the 2(-Delta Delta C(T)) method. Methods. 2001;25:402–408. 11846609 10.1006/meth.2001.1262

[21] Zhao F, Chen CW, Yang WW, et al. Hsa_circRNA_0059655 plays a role in salivary adenoid cystic carcinoma by functioning as a sponge of miR-338-3p. Cell Mol Biol (Noisy-le-grand). 2018;64:100–106. 30672444

[22] Panda AC. Circular RNAs act as miRNA sponges. Adv Exp Med Biol. 2018;1087:67–79. 30259358 10.1007/978-981-13-1426-1_6

[23] Gokul S, Rajanikant GK. Circular RNAs in brain physiology and disease. Adv Exp Med Biol. 2018;1087:231–237. 30259370 10.1007/978-981-13-1426-1_18

[24] Hao Z, Hu S, Liu Z, et al. Circular RNAs: functions and prospects in glioma. J Mol Neurosci. 2019;67:72–81. 30460608 10.1007/s12031-018-1211-2

[25] Li G, Yang H, Han K, et al. A novel circular RNA, hsa_circ_0046701, promotes carcinogenesis by increasing the expression of miR-142-3p target ITGB8 in glioma. Biochem Biophys Res Commun. 2018;498:254–261. 29337055 10.1016/j.bbrc.2018.01.076

[26] Jin P, Huang Y, Zhu P, et al. CircRNA circHIPK3 serves as a prognostic marker to promote glioma progression by regulating miR-654/IGF2BP3 signaling. Biochem Biophys Res Commun. 2018;503:1570–1574. 30057315 10.1016/j.bbrc.2018.07.081

[27] Duan X, Liu D, Wang Y, et al. Circular RNA hsa_circ_0074362 promotes glioma cell proliferation, migration, and invasion by attenuating the inhibition of miR-1236-3p on HOXB7 expression. DNA Cell Biol. 2018;37:917–924. 30388035 10.1089/dna.2018.4311

[28] Xia B, Hong T, He X, et al. A circular RNA derived from MMP9 facilitates oral squamous cell carcinoma metastasis through regulation of MMP9 mRNA stability. Cell Transplant. 2019;963689719875409. DOI:10.1177/0963689719875409 PMC6923549 31510782

[29] Pan G, Hu T, Chen X, et al. Upregulation of circMMP9 promotes osteosarcoma progression via targeting miR-1265/CHI3L1 axis. Cancer Manag Res. 2019;11:9225–9231. 31754311 10.2147/CMAR.S226264 PMC6825504

[30] Zhou X, Tao H. Overexpression of microRNA-936 suppresses non-small cell lung cancer cell proliferation and invasion via targeting E2F2. Exp Ther Med. 2018;16:2696–2702. 30210611 10.3892/etm.2018.6490 PMC6122560

[31] Li C, Yu S, Wu S, et al. MicroRNA-936 targets FGF2 to inhibit epithelial ovarian cancer aggressiveness by deactivating the PI3K/Akt pathway. Onco Targets Ther. 2019;12:5311–5322. 31371979 10.2147/OTT.S213231 PMC6626896

[32] Li R, Wu B, Xia J, et al. Circular RNA hsa_circRNA_102958 promotes tumorigenesis of colorectal cancer via miR-585/CDC25B axis. Cancer Manag Res. 2019;11:6887–6893. 31413634 10.2147/CMAR.S212180 PMC6662515

[33] Sun X, Ge X, Xu Z, et al. Identification of circular RNA-microRNA-messenger RNA regulatory network in hepatocellular carcinoma by integrated analysis. J Gastroenterol Hepatol. 2020;35:157–164. 31222831 10.1111/jgh.14762

[34] Lu C, Shi X, Wang AY, et al. RNA-Seq profiling of circular RNAs in human laryngeal squamous cell carcinomas. Mol Cancer. 2018;17:86. 29716593 10.1186/s12943-018-0833-x PMC5930968

[35] Tong H, Zhao K, Wang J, et al. CircZNF609/miR-134-5p/BTG-2 axis regulates proliferation and migration of glioma cell. J Pharm Pharmacol. 2020;72:68–75. 31721211 10.1111/jphp.13188

[36] Lei B, Huang Y, Zhou Z, et al. Circular RNA hsa_circ_0076248 promotes oncogenesis of glioma by sponging miR-181a to modulate SIRT1 expression. J Cell Biochem. 2019;120:6698–6708. 30506951 10.1002/jcb.27966 PMC6587862

[37] Wang J, Zhu W, Tao G, et al. Circular RNA circ-LRP6 facilitates Myc-driven tumorigenesis in esophageal squamous cell cancer. Bioengineered. 2020;11:932–938. 32867570 10.1080/21655979.2020.1809922 PMC8291805

[38] Gu Q, Hou W, Shi L, et al. Circular RNA ZNF609 functions as a competing endogenous RNA in regulating E2F transcription factor 6 through competitively binding to microRNA-197-3p to promote the progression of cervical cancer progression. Bioengineered. 2021;12:927–936. 33734009 10.1080/21655979.2021.1896116 PMC8291891

[39] Gao Q, Liu Q, Chen H. Circular RNA hsa_circ_0000117 accelerates the proliferation and invasion of gastric cancer cells by regulating the microRNA-337-3p/signal transducer and activator of transcription 3 axis. Bioengineered. 2021;12:1381–1390. 33896365 10.1080/21655979.2021.1918992 PMC8806281

