# Supplementary material for: Circular RNA circ_0001162 promotes cell proliferation and invasion of glioma via the miR-936/ERBB4 axis
Source: Bioengineered. 2021 May 30;12(1):2106–18. doi: 10.1080/21655979.2021.1932221 (PMC8806513; doi:10.1080/21655979.2021.1932221)
Supplement: Supplemental Material [file KBIE_A_1932221_SM2338.zip › supplementary figures.pdf]

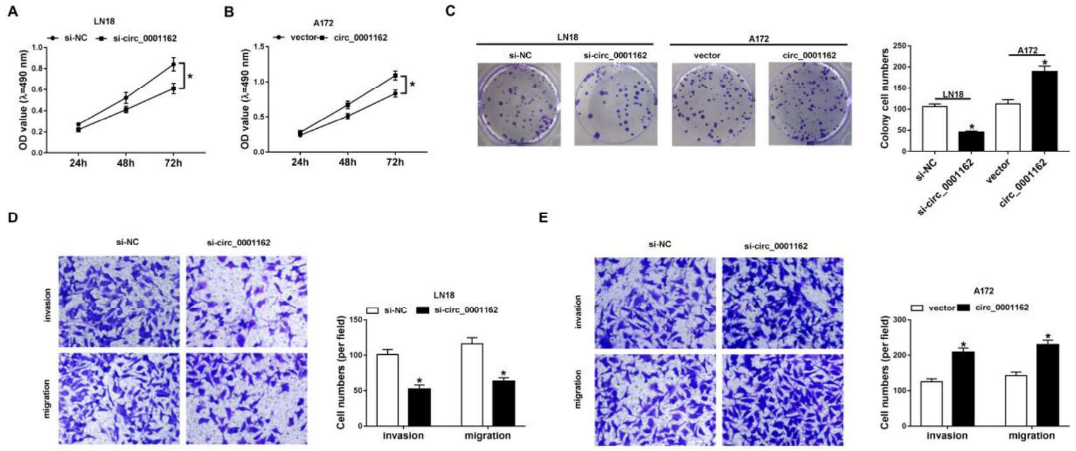

Supplementary Fig.1. Circ_0001162 knockdown in LN18 cells and circ_0001162 overexpression in A172 cells suggested that circ_0001162 promoted glioma progression. LN18 cells were transfected with si-NC or si-circ_0001162 and A172 cells were transfected with vector or circ_0001162. (A-B) Cell proliferation was analyzed using MTT assay. (C) Colony formation capacity was detected using colony formation assay. (D-E) Cell migration and invasion were assessed by transwell assay. *P< 0.05.

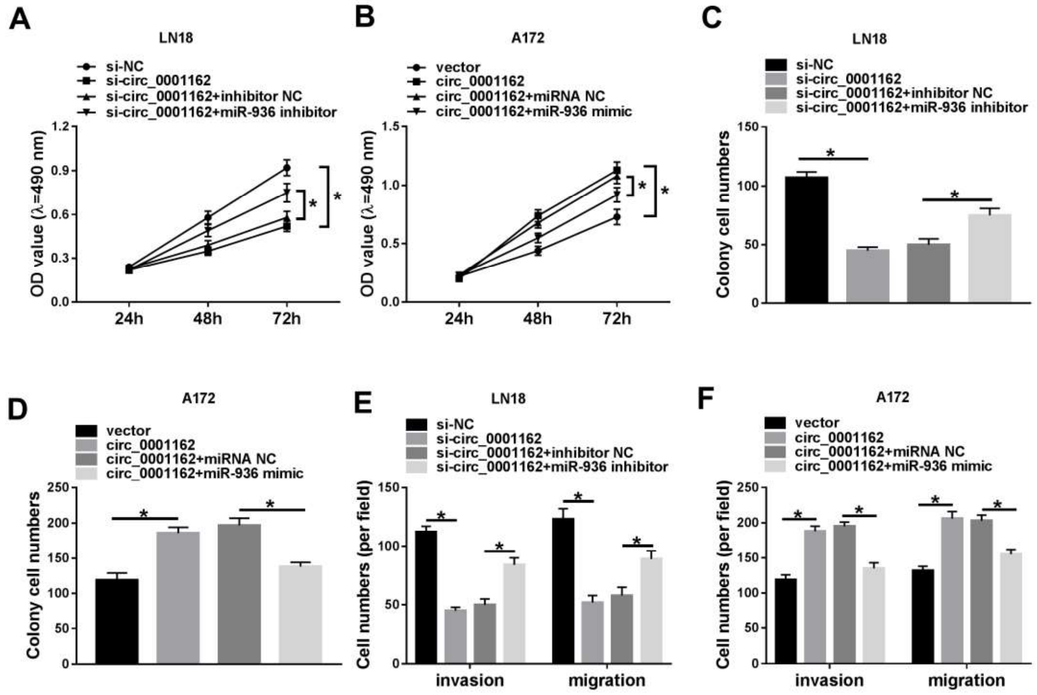

Supplementary Fig.2. Circ_0001162/miR-936 axis was identified in LN18 and A172 cells. LN18 cells were transfected with si-NC, si-circ_0001162, si-circ_0001162+inhibitor NC or si-circ_0001162+miR-936 inhibitor and A172 cells were transfected with vector, circ_0001162, circ_0001162+miRNA NC or circ_0001162+miR-936 mimic. (A-B) MTT assay was adopted for proliferation detection. (C-D) Colony formation assay was used for colony formation analysis. (E-F) Transwell assay was applied for cell migration and invasion determination. *P< 0.05.

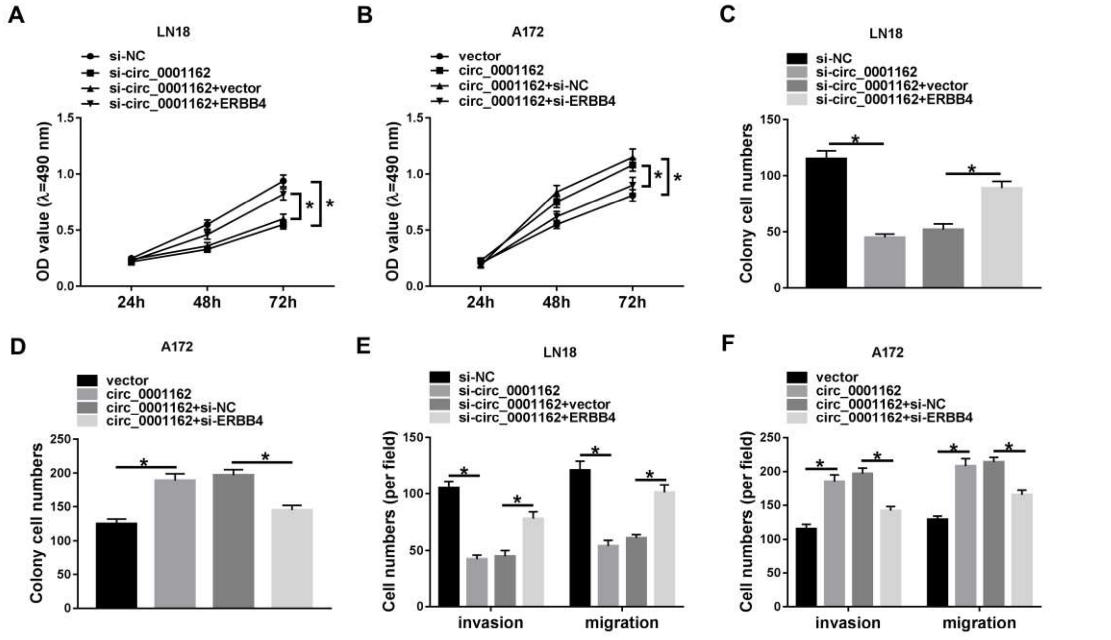

Supplementary Fig.3. Circ_0001162 knockdown in LN18 cells and circ_0001162 overexpression in A172 cells suggested that circ_0001162 promoted glioma progression. LN18 cells were transfected with si-NC, si-circ_0001162, si-circ_0001162+vector or si-circ_0001162+ERBB4 and A172 cells were transfected with vector, circ_0001162, circ_0001162+si-NC or circ_0001162+si-ERBB4. (A-B) Cell proliferation examination was performed through MTT assay. (C-D) Colony formation ability was assessed via colony formation assay. (E-F) Cell migration and invasion evaluation was carried out using transwell assay. *$P < 0.05$.
